# Serum Iodine and Bromine in Chronic Hemodialysis Patients—An Observational Study in a Cohort of Portuguese Patients

**DOI:** 10.3390/toxics11030247

**Published:** 2023-03-06

**Authors:** Gergana Novakova, Presian Bonev, Mary Duro, Rui Azevedo, Cristina Couto, Edgar Pinto, Agostinho Almeida

**Affiliations:** 1 LAQV/REQUIMTE, Department of Chemical Sciences, Faculty of Pharmacy, University of Porto, 4050-313 Porto, Portugal; 2 FP-ENAS—Fernando Pessoa Energy, Environment and Health Research Unit, Fernando Pessoa University, 4249-004 Porto, Portugal; 3 Laboratório de Análises Clínicas Dra. Matilde Sampaio, 5200-216 Mogadouro, Portugal; 4 Laboratório de Análises Clínicas Vale do Sousa, 4560-547 Penafiel, Portugal; 5 TOXRUN—Toxicology Research Unit, University Institute of Health Sciences, CESPU, 4585-116 Gandra, Portugal; 6 Department of Environmental Health, ESS, Polytechnic of Porto, 4200-072 Porto, Portugal

**Keywords:** iodine, bromine, serum levels, ERSD, hemodialysis patients

## Abstract

**Background**: Patients on chronic hemodialysis therapy are at high risk of disturbances in trace element status due to both the underlying disease and the hemodialysis process itself. Data on iodine and bromine levels in these patients are scarce. **Methods**: Using an ICP-MS analytical procedure, serum iodine and bromine levels were determined in a cohort (n = 57) of end-stage renal disease patients on chronic hemodialysis. The results were compared with those of a control group (n = 59). **Results**: Hemodialysis patients presented serum iodine levels within the normal range, slightly lower than in controls, but without reaching a statistically significant difference (67.6 ± 17.1 µg/L vs. 72.2 ± 14.8 µg/L; *p* = 0.1252). In contrast, serum bromine levels were much lower in patients (1086 ± 244 µg/L vs. 4137 ± 770 µg/L; *p* < 0.0001), at values only about 26% of the values observed in controls. **Conclusions**: Hemodialysis patients had normal serum iodine levels, but highly decreased serum bromine levels. The clinical significance of this finding requires further investigation, but it may be associated with sleep disturbances and fatigue that affect hemodialysis patients.

## 1. Introduction

Developed in the second half of the 20th century, modern hemodialysis therapy became the treatment of choice for acute and chronic renal failure and, since then, has made it possible to prolong the lives of millions of people around the world [1]. In practical terms, a machine pumps the patient’s venous blood to a dialyzer (“artificial kidney”) where it is cleared through a semi-permeable membrane (which separates the blood from the dialysate: the acceptor fluid), and then returns it to the patient’s circulatory system. The basic principle of the dialysis process is the diffusion of solutes through a semipermeable membrane according to an electrochemical concentration gradient [1,2]. When applied to blood (hemodialysis), the main purpose is to eliminate metabolic waste products (e.g., urea, creatinine), which diffuse from the blood plasma into the dialysate (an aqueous electrolyte solution that acts as an acceptor fluid). In addition, through a process called ultrafiltration, excess water is also removed from the body [1,3]. The ultimate goal is to restore intracellular and extracellular homeostasis, artificially performing those functions that under normal conditions would be performed by the kidneys. In addition to the concentration gradient, the diffusion rate of solutes from blood plasma to dialysate across the semipermeable membrane is determined in an inverse way by the particle size of the solute [2]. Thus, ions (e.g., Na^+^, K^+^, Mg^2+^, Cl^−^) and small molecules (such as urea, creatinine, glucose) diffuse rapidly, while larger molecules (e.g., albumin) and protein-bound solutes diffuse much more slowly [1,3].

Trace elements are defined as chemical elements present in body fluids and tissues in small amounts. According to a widely accepted definition [4], trace elements are those present in human body fluids, such as blood plasma, at levels below 10 mg/L. Several trace elements are known to be physiologically essential (e.g., zinc, copper, selenium, iodine), as they are required for various biochemical processes. Others are eminently toxic, causing harmful effects even at very low concentrations (e.g., lead, cadmium, mercury), and others may be beneficial, or even essential, but there is still not enough evidence for them to be considered as such, as is the case with bromine [5].

Hemodialysis patients are at high potential risk of trace elements deficiency or excess [6]. On the one hand, they are exposed to large volumes of dialysate (300–600 L per week) [7], which puts them at great risk of systemic overload if the required quality of dialysate is not guaranteed—an issue that has received considerable attention following the recognition in the late 1970s that some serious complications in hemodialysis patients (anemia, encephalopathy, osteodystrophy) were associated with aluminum overload and toxicity [8,9,10]. On the other hand, due to the intrinsic nature of the process, hemodialysis does not just remove toxins and excess body fluids, it can also cause the loss of physiologically important substances, including essential trace elements [8,9,10,11].

In a previous study, we reported serum levels of a wide panel of trace metals (Li, Al, Mn, Co, Ni, Cu, Zn, Rb, Sr, Mo, Cd, Ba, Pb) and Se in a cohort of hemodialysis patients [12]. Here, we present data on serum levels of iodine and bromine, which is an understudied subject.

## 2. Materials and Methods

### 2.1. Samples

Patient group (n = 57)—Serum samples were obtained from patients undergoing hemodialysis therapy at a dialysis center in northeastern Portugal. The patients underwent hemodialysis 3 times a week (Monday, Wednesday, and Friday). The time on dialysis was 9.5 ± 8.2 years. Samples were collected as part of patients’ routine laboratory testing, during the day, prior to the hemodialysis session (non-fasting conditions). The subjects’ mean age was 68.9 ± 14.0 years, and they were 57.9% male and 42.1% female.

Control group (n = 59)—For comparison purposes, serum samples were obtained from individuals with no evidence of renal disease according to standard clinical laboratory criteria who attended the same clinical laboratory for routine analysis. Their mean age was 57.4 ± 17.9 years, and they were 47.5% male and 52.5% female.

Blood samples were collected by conventional venipuncture into VACUETTE^®^ Z Trace Elements No Additive Blood Collection Tubes (Greiner Bio-One, Kremsmünster, Austria). After allowing the blood to clot for 30 min at room temperature, the tubes were centrifuged at 1300 g for 10 min, and the serum was separated into acid washed Eppendorf tubes and stored at 4 ℃ until analysis.

### 2.2. Chemicals, Labware, and Standard Solutions

Ultrapure water (resistivity >18.2 MΩ.cm at 25 °C) produced in an Aarium^®^ pro water purification system (Sartorius, Gottingen, Germany) was used. Tetramethylammonium hydroxide (TMAH) solution (25% *w*/*w* in H_2_O) and Triton X-100 (laboratory grade) were obtained from Sigma-Aldrich (Buchs, Switzerland).

All laboratory ware (bottles, tubes, volumetric flasks) was made of polypropylene and was decontaminated by immersion in a 2% (*v*/*v*) TMAH solution for at least 24 h, followed by thorough rinsing with ultrapure water and drying at room temperature under dust-free conditions.

Iodide Standard for ICP (TraceCERT^®^, 1000 mg/L I^−^ in water, from Sigma-Aldrich) and Bromide ICP Standard (AccuSPEC, 1000 mg/L Br^−^ in water, from SCP Science, Baie-D’Urfe, Quebec, Canada) were used to prepare the calibration standards. Tellurium Standard for ICP (TraceCERT^®^, 1000 mg/L Te, Sigma-Aldrich) was used to prepare the internal standard (IS) solution.

Calibration standard solutions (n = 5) were prepared in the range of 200–20,000 µg/L for bromine and 20–200 µg/L for iodine by properly diluting commercial stock solutions with ultrapure water.

### 2.3. ICP-MS Instrument

Determination of iodine and bromine in serum samples was performed using an iCAP™ Q ICP-MS instrument (Thermo Fisher Scientific, Bremen, Germany). It was equipped with a Meinhard^®^ (Golden, CO) TQ+ quartz concentric nebulizer, a Peltier cooled high-purity quartz baffled cyclonic spray chamber, and a quartz torch with a 2.5 mm ID quartz injector. The interface with the mass spectrometer consisted of a two-nickel cone (sample and skimmer) system.

High-purity argon (99.9997%, from Gasin, Leça da Palmeira, Portugal) was used as nebulizer and plasma gas. The instrument was tuned daily for maximum sensitivity and signal stability, and for minimum production oxides and doubly charged ions. Typical operating conditions of the ICP-MS instrument are shown in Table S1 (Supplementary Material).

### 2.4. Analytical Procedure

Determination of iodine and bromine was performed based on a Centers for Disease Control and Prevention (CDC) procedure [13]. Briefly, calibration standards, study samples, and control samples were diluted 1:10 with an aqueous solution containing 1% (*v*/*v*) TMAH, 0.01% (*v*/*v*) Triton X-100, and 100 µg/L Te (IS). After complete homogenization on a vortex mixer, the solutions were presented to the ICP-MS instrument using a CETAC ASX-520 autosampler (Teledyne CETAC Technologies, Omaha, NE, USA). Iodine, bromine, and tellurium (IS) were measured at m/z ratios 127, 81, and 125, respectively.

### 2.5. Analytical Quality Control

Analytical accuracy was assessed by repeated analysis (at the beginning, middle, and end of the analytical run) of Seronorm™ Trace Elements Serum L-1 and L-2, purchased from SERO AS (Billingstad, Norway). The results are shown in Table S2 (Supplementary Material). To control for memory and carryover effects, calibration blanks were run periodically.

### 2.6. Statistical Data Analysis

Instrumental data acquisition and data processing was performed using Thermo Scientific™ Qtegra™ Intelligent Scientific Data Solution™ (ISDS) platform software (v. 2.10, Thermo Fisher Scientific). Statistical analysis was performed using Microsoft Excel 2016 (Windows version). Iodine and bromine concentrations in serum samples were expressed as mean ± standard deviation (SD), median, and range. Comparison between groups was performed using the Student’s *t*-test. Statistical significance was considered for *p* < 0.05.

## 3. Results

Serum samples from a total of 57 individuals in the patient group (individuals on chronic hemodialysis therapy) and 59 individuals in the control group (individuals with normal renal function according to laboratory data) were analyzed for their total iodine and bromine concentration. Results (µg/L) are summarized in Table 1. One sample from the patients group was excluded for presenting a very high concentration (“outlier”) of iodine (2065 µg/L), which could be due to a patient’ radiographic examination in the days prior to blood sample collection, as iodine is a constituent of commonly used contrast agents [14].

Serum iodine levels were well within the reference ranges adopted by large international clinical laboratories: 40.0–92.0 µg/L [15,16,17]; 52–109 µg/L [18] in both patient and control groups. The mean value was higher in controls than in patients (72.2 ± 14.8 µg/L vs. 67.6 ± 17.1 µg/L), but the difference did not reach statistical significance according to a Student’s *t*-test at 95% confidence level (*p* = 0.1252).

For bromine, a statistically highly significant difference was observed between the two groups, with much lower values in the patient group: 1086 ± 244 µg/L vs. 4137 ± 770 µg/L (*p* < 0.0001). There is no well-defined reference interval for bromine. The population reference range derived from data from a large laboratory (2.5th–97.5th percentiles) has been established at 0.9–7.3 mg/L [19], but most literature reports values between 3 and 7 mg/L [20,21,22,23,24,25]. It can therefore be concluded that serum bromine levels were normal in the control group and significantly decreased in the patient group.

## 4. Discussion

Iodine is a well-recognized essential trace element. It is required for the production of the thyroid hormones thyroxine (T4) and triiodothyronine (T3), which regulate numerous metabolic activities and physiologic processes, including general body growth, neurological development, and reproductive function [26].

Iodine is required at levels of 150 µg/day in adult men and women, with higher amounts being required during pregnancy and lactation [27]. Important dietary sources include dairy products, marine fish, and shellfish, as well as cereals and grains, but the contents vary greatly depending on the amount of iodine in the soil [27,28].

Ingested orally (either as soluble iodide or iodate salts), iodine is virtually 100% absorbed from the gastrointestinal tract [27,29] and reaches the plasma iodine pool, from where it can be taken up by the thyroid gland (20–30%) or eliminated by the kidneys (30–60%) in approximately 10 h [27,29]. Urinary clearance is fairly constant, but thyroid uptake varies inversely with iodine intake (the percentage of thyroid uptake increases with decreasing levels of iodine intake) [28].

The total iodine content of the human body is approximately 10–15 mg, most of which (approximately 70–90%) is found in the thyroid gland [29]. In blood plasma, the concentration of iodine is usually in the range of 50–100 µg/L, the vast majority (95%) being present as various organic forms of iodine (mostly T4 and T3 protein complexes), and only about 5% in the inorganic form (iodide) [29].

Because it is a key component of thyroid hormones, iodine deficiency is associated with an increased risk of hypothyroidism, as observed worldwide in populations residing in regions with low soil iodine content [26,28].

In adults, the spectrum of iodine deficiency disorders includes goiter (with its complications), hypothyroidism (in mild to severe iodine deficiency), impaired mental function, spontaneous hyperthyroidism in the elderly, and iodine-induced hyperthyroidism [26,30]. However, through several complex mechanisms, in certain susceptible individuals (including those with pre-existing thyroid disease, patients with other risk factors, and the elderly), excess iodine (i.e., supraphysiological exposure to iodine) can also affect thyroid function, causing both hypothyroidism and hyperthyroidism, either subclinical or overt [26,31,32].

Dietary intake of iodine is usually assessed by measuring urinary iodine concentration (UIC). However, the large daily variation in UIC makes it difficult to quantify the actual individual iodine intake [33,34].

Serum iodine concentrations have shown a strong nonlinear correlation with UIC and thyroid function [35,36] and may be a better index of individual dietary iodine intake and iodine overload. In a study with a pool of serum samples (n = 30) for which T3 and T4 levels were available, we found that they were highly correlated (r = 0.910) with total iodine concentration as determined by ICP-MS (Figure S1, Supplementary Material).

A serum iodine level greater than 100 µg/L has been considered a risk factor for thyroid disease [35].

The most common complication in chronic kidney disease related to thyroid function is subclinical hypothyroidism, and its prevalence increases consistently with the decline in glomerular filtration rate [37]. In chronic renal failure, decreased urinary iodine clearance increases serum levels of inorganic iodine (iodide) [38] and thyroid iodine content, which can potentially block thyroid hormone production (the Wolff–Chaikoff effect) [39] and enlarge the gland [40]. That is, in patients with chronic renal failure, hypothyroidism and goiter can be induced by excess iodine due to decreased renal iodide excretion [41].

In our study, serum iodine levels (total iodine, as measured by ICP-MS) were well within the widely accepted reference range (“normal values”) of 50–100 µg/L [29]. The mean value was higher in controls than in patients (72.2 ± 14.8 µg/L vs. 67.6 ± 17.1 µg/L), but the difference did not reach statistical significance. This means that the hemodialysis process, which is even used to treat acute toxic plasma iodine levels [42], efficiently removes iodide from blood plasma (thyroid hormone losses during hemodialysis are insignificant [41]) and maintains plasma levels at appropriate values.

As total iodine was determined (ICP-MS measures the total concentration of the element in the sample), lower serum values in patients may also be related to lower levels of T3 and T4, as both tend to be reduced in chronic renal failure [41].

Lower iodine levels in hemodialysis patients compared to controls may also be related to reduced dietary iodine intake, mainly due to dietary restriction of milk and dairy products [43], major sources of iodine in Western diets [44].

Bromine, an element of the halide family, as well as iodine, chlorine, and fluorine, is one of the most abundant and ubiquitous trace elements in the biosphere, widely distributed across mammalian tissues as the bromide anion (Br^−^) [45,46].

In the human body, it is present in significant amounts (ca. 200 mg [47] versus just 10–20 mg of iodine [26]), with a blood concentration ranging between 3.2 and 5.6 mg/L [47]. The estimated daily dietary intake of bromide is approximately 2–8 mg, with the main sources being marine foods (fish, shellfish, and seaweed), grains, and nuts [47].

Dietary bromine is well absorbed from the gastrointestinal tract and is excreted primarily in the urine [48], making urinary bromine concentration a good indicator of daily intake [23].

Due to its structural similarity, bromine may compete with iodine for uptake by the thyroid gland. Under normal conditions, this effect is negligible, but under circumstances of low iodine and high bromine levels, there may be decreased thyroid hormone synthesis [48,49].

Bromine has been considered a “possibly essential element” because, despite circumstantial evidence of essentiality, no specific biochemical function in higher animals has been recognized [50]. However, there is growing evidence for the essentiality of bromine [47]. Specifically, McCall et al. [46] unraveled the missing evidence to declare bromine essential for animals, including the mechanistic biochemical explanation of its physiological role: bromide was shown to be necessary for the assembly of cross-linked collagen IV scaffolds, which are crucial for tissue development in animals. The authors even anticipated the implications that increased bromine losses during hemodialysis might have in end-stage renal disease patients, who may have impaired tissue development and remodeling due to bromine deficiency.

Studies on serum bromine levels in hemodialysis patients are very scarce. Miura et al. [51] found serum concentrations of bromine in hemodialysis patients of 1702 ± 240 µg/L, significantly lower (*p* < 0.0001) than those of normal controls (5530 ± 663 µg/L). In contrast, the mean serum concentration in patients with kidney disease but not on hemodialysis was significantly higher: 6544 ± 927 µg/L (*p* < 0.001). Later, the same group showed that serum bromine levels decreased significantly (from 1662 ± 367 µg/L pre-dialysis to 959 ± 112 µg/L post-dialysis) and dialysate bromine levels increased significantly during the hemodialysis session, showing that bromine is efficiently transferred from serum to dialysis fluid during the hemodialysis process [52].

In our study, a marked difference in serum bromine levels between hemodialysis patients and controls was also found: 1086 ± 244 µg/L vs. 3137 ± 770 µg/L (*p* < 0.0001), respectively. There was no correlation between bromine concentration and patient age (R^2^ = 0.009) or time on dialysis therapy (R^2^ = 0.011).

The actual clinical impact of this bromine “deficiency” is unknown. The most interesting issue seems to be the possible relationship with the sleep disorders that affect patients on chronic hemodialysis.

Estimates suggest that 40–85% of hemodialysis patients have sleep disturbances, especially insomnia [53]. This has been ranked as one of the major threats to patients’ quality of life and has the potential to lead to other complications, including depression, decreased immune response, and increased cardiovascular risk [53].

Interestingly, the possible association between bromine deficiency and insomnia in hemodialysis patients was suggested more than 40 years ago, but later seems to have been forgotten [54]. Many years later (in 2006), Canavese et al. [55] raised the question again, noting that bromine is associated with brain metabolism, is used as a sedative to induce sleep, and increases in animals during hibernation as well as in sleeping humans, further highlighting the discovery of a bromine compound with REM-sleep-inducing and anti-cholinesterase activities that was isolated from human cerebrospinal fluid and identified as 1-methylheptyl gamma-bromoacetoacetate The authors concluded with a strong suggestion “that further studies address the evaluation of bromine status in dialysis patients”, which was what we intended in this study. Future studies should address another recommendation by Canavese et al., that of assessing whether supplementation, when indicated, may contribute to the correction of sleep disturbances that affect patients on hemodialysis [55].

## 5. Conclusions

This study confirms that hemodialysis patients tend to have significant trace element imbalances compared to individuals with normal renal function.

As far as we can see in the literature, this seems to be the first report of serum total iodine levels in hemodialysis patients. The results showed normal values, only slightly lower than those of the control group, which could be due to the removal of iodide from the plasma during the hemodialysis process, the hypothyroidism that affects some patients on hemodialysis, or a reduced intake of iodine due to patients’ dietary restrictions.

In contrast, serum bromine levels were only about 25% of those in the control group. The possible essentiality of bromine is still an open question, but this significant decrease in bromine levels in hemodialysis patients may be associated with some disorders that affect them, including impaired tissue development and remodeling, but especially insomnia. As emphasized above, further studies addressing this issue are needed.

If bromine is truly an essential element, it should follow (like the other essential elements) a physiological effect vs. dose (or blood concentration) according to the Bertrand diagram.

The toxic effects of excess bromine (right part of the Bertrand diagram) are well known. Until the appearance of modern antiepileptics (e.g., carbamazepine, valproate, lamotrigine), bromine salts were widely used as hypnotic sedatives and in the treatment of convulsive seizures [56], so there is ample information on the toxic effects of bromine. Intoxication, called “bromism”, is accompanied by predominantly neurological manifestations (lethargy, tremor, dysarthria, ataxia), psychiatric disorders (such as delusions and hallucinations), and dermatological manifestations (skin eruptions or acne-forming dermatitis) [56].

It remains to be seen what happens on the left side of the Bertrand diagram, i.e., under circumstances of “deficiency” (blood levels chronically below normal). People on chronic hemodialysis, because they have extraordinarily low levels of bromine (bromide), as documented here, removed from the bloodstream during the dialysis process, represent an excellent experimental model to study this question. If bromine supplementation, to bring serum levels within normal limits, somehow reversed the symptoms presented by patients, it would make an important contribution to clarifying the question of whether or not bromine is physiologically essential.

## Figures and Tables

**Table 1:** Summary of the results: serum iodine and bromine levels (µg/L) in patients on chronic hemodialysis therapy and in a control group.

	Iodine		Bromine	
	Patients	Controls	Patients	Controls
N	56	59	57	59
Mean ± SD	67.6 ± 17.1	72.2 ± 14.8	1086 ± 244	4137 ± 770
Min–Max	42.3–107.4	49.7–113.4	810–2515	3056–7371

## Data Availability

Details about the data presented in this study are available on request from the corresponding author. The data are not publicly available due to privacy and ethical restrictions.

## Supplementary Materials

The following supporting information can be downloaded at https://www.mdpi.com/article/10.3390/toxics11030247/s1: Table S1: ICP-MS instrument (iCAP™ Q, Thermo Scientific) operating parameters; Table S2: Summary of quality control results; Figure S1: Relationship between total iodine in serum (µg/L) as determined by ICP-MS) and T3 + T4 (in µg/L, as determined by electrochemiluminescence immunoassays) (n = 30 serum samples).
