# Supplementary material for: Serum Iodine and Bromine in Chronic Hemodialysis Patients—An Observational Study in a Cohort of Portuguese Patients
Source: Toxics. 2023 Mar 6;11(3):247. doi: 10.3390/toxics11030247 (PMC10053604; doi:10.3390/toxics11030247)
Supplement: Supplementary file 1 [file toxics-11-00247-s001.zip › toxics-2249678-supplementary.pdf]

Supplementary Material

Serum Iodine and Bromine in Chronic Hemodialysis Patients—An Observational Study in a Cohort of Portuguese Patients

Gergana Novakova [1], Presian Bonev [1], Mary Duro [1,2,3], Rui Azevedo [1], Cristina Couto [1,4], Edgar Pinto [1,5] and Agostinho Almeida [1,*]

Table S1: ICP-MS instrument (iCAP™ Q, Thermo Scientific) operating parameters.

**Instrumental parameter**	**Operating conditions**
RF power	1550 W
Plasma gas (Ar) flow	14.0 L/min
Nebulizer gas flow	1.17 L/min
Auxiliary gas flow	0.79 L/min
Spray chamber temperature	2.6 °C
Dwell time	10 ms
Replicates	3

Table S2. Summary of Quality Control results.

Element	QC sample	Analytical run*	Obtained value, mean (±SD)	Informative value
**Iodine**	L-1	1 (n=3)	**67.0** (±1.1)	**66**
		2 (n=2)	**62.4** (±0.05)	
	L-2	1 (n=3)	**60.1** (±3.0)	**60.9**
		2 (n=3)	**68.5** (±3.0)	**69**
**Bromine**	L-1	1 (n=3)	**686.2** (±7.8)	**709**
		2 (n=2)	**687.9** (±4.3)	
	L-2	1 (n=2)	**766.0** (±4.4)	**773**
		2 (n=2)	**769.6** (±34.5)	**753**

L-1: Seronorm™ Trace Elements Serum, lot 1801802. L-2: Seronorm™ Trace Elements Serum, lot 1309416 and lot 1801803. * n = number of determinations within the analytical run.

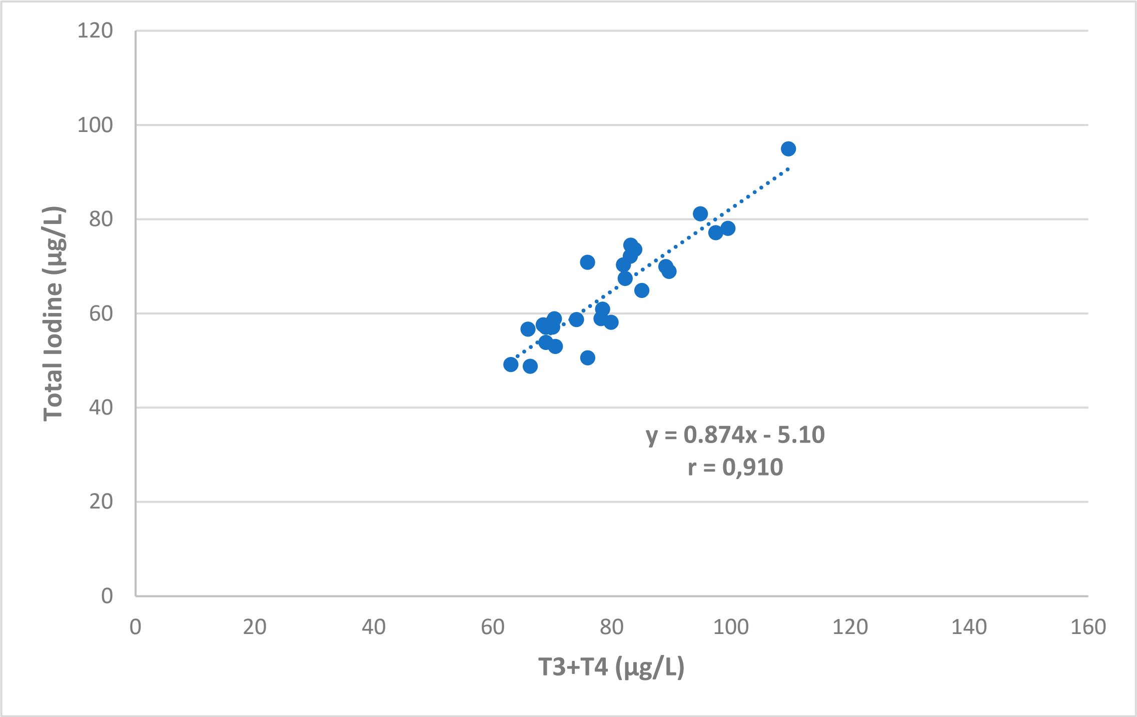

Figure S1: Relationship between total iodine in serum (µg/L) as determined by ICP-MS) and T3+T4 (in µg/L, as determined by electrochemiluminescence immunoassays) (n=30 serum samples).
